# Iron Pyrite/Titanium Dioxide Photoanode for Extended Near Infrared Light Harvesting in a Photoelectrochemical Cell

**DOI:** 10.1038/srep20397

**Published:** 2016-02-08

**Authors:** Di-Yan Wang, Cheng-Hung Li, Shao-Sian Li, Tsung-Rong Kuo, Chin-Ming Tsai, Tin-Reui Chen, Ying-Chiao Wang, Chun-Wei Chen, Chia-Chun Chen

**Affiliations:** 1 Department of Chemistry, National Taiwan Normal University, Taipei 11677, Taiwan; 2 Department of Materials Science and Engineering, National Taiwan University, Taipei 10617, Taiwan; 3 Institute of Atomic and Molecular Sciences, Academia Sinica, Taipei 10617, Taiwan

## Abstract

The design of active and stable semiconducting composites with enhanced photoresponse from visible light to near infrared (NIR) is a key to improve solar energy harvesting for photolysis of water in photoelectrochemical cell. In this study, we prepared earth abundant semiconducting composites consisting of iron pyrite and Titanium oxide as a photoanode (FeS_2_/TiO_2_ photoanode) for photoelectrochemical applications. The detailed structure and atomic compositions of FeS_2_/TiO_2_ photoanode was characterized by high-resolution transmission electron microscopy (HRTEM), energy-dispersive X-ray spectroscopy (EDS), powder X-ray diffraction (XRD), inductively coupled plasma with atomic emission spectroscopy (ICPAES) and Raman spectroscopy. Through the proper sulfurization treatment, the FeS_2_/TiO_2_ photoanode exhibited high photoresponse from visible light extended to near infrared range (900 nm) as well as stable durability test for 4 hours. We found that the critical factors to enhance the photoresponse are on the elimination of surface defect of FeS_2_ and on the enhancement of interface charge transfer between FeS_2_ and TiO_2_. Our overall results open a route for the design of sulfur-based binary compounds for photoelectrochemical applications.

Solar-induced water splitting by photoelectrochemical (PEC) cells provides an ideal solution to generate hydrogen energy, which is derived by electrochemical photolysis of H_2_O with semiconductors as photoanode and photocathode materials^1^,^2^,^3^. The effectiveness of photo-driven electrolysis processes showed strong dependency on the capability of absorbing UV, visible and infrared (UV-vis-NIR) light of semiconductors, as well as their ability to suppress the rapid combination of photogenerated electrons and holes^4^,^5^. Titanium dioxide (TiO_2_) has been considered to one of most attractive materials for PEC application because of its high photocatalytic activity and excellent chemical stability in the strong alkaline solution^6^,^7^,^8^. However, the absorption spectrum of TiO_2_ with large band gap (~3.2 eV) is only located on UV light (5% of sunlight), which cause less energy conversion efficiency. Recently, researchers have paid attention on finding the solutions to extend absorption range of TiO_2_ to visible light for enhancing light harvesting ability. An efficient method to narrow the band gap of TiO_2_ was utilizing chemical doping^9^,^10^,^11^ or increasing of defect states^12^,^13^ in TiO_2_ crystal structure. For example, a study indicated that the band gap of TiO_2_ was successfully reduced to 1.53 eV (absorption spectrum extend to ~810 nm) by introducing disorder in the surface layers of TiO_2_ through hydrogenation^12^. Although chemical doping TiO_2_ exhibited a great optical response to solar radiation, its absorption range in the visible and infrared remains insufficient^9^.

The way to extend light harvesting of TiO_2_ photoanode from visible and even near infrared (NIR) range is sensitizing lower band-gap chalcogenide semiconductors on TiO_2_, such as CdS^14^,^15^, CdSe^16^, and PbS^17^,^18^. The approaches have been widely applied in quantum-dot sensitized solar cells (QDSSCs)^19^,^20^ and photoelectrochemical cell^21^. The advantages of these chalcogenides materials are their low band gaps (CdS~ 2.4 eV, CdSe~1.7 eV and PbS~1 eV) and efficient charge transfer from the chalcogenides to TiO_2_ due to their type II electronic band structure^15^. For examples, the N doping of TiO_2_ nanowires sensitized by CdSe as the photoanode in PEC resulted in photocurrents close to 3 mA·cm^−2^ 22. Other reports have also highlighted the importance of the controlled deposition of the light-absorbing semiconductor (CdSe) on inverse opals of TiO_2_, resulting in photocurrents of 15.7 mA·cm^−2^ under AM 1.5 illumination^23^. However, Both Cd and Pb elements are considered to be quite toxic^24^. Therefore, searching low-cost and environmental-friendly materials as alternatives to toxic metal is crucial to make PEC more competitive for future commercial applications.

Earth-abundance and non-toxicity pyrite iron disulfide (FeS_2_) is a potential candidate to be applied for next-generation photovoltaic because it’s large optical absorption coefficient (>10^5 ^cm^−1^) and a narrow band gap of 0.95 eV^25^,^26^. FeS_2_ has been predicted as showing the highest material availability among 23 existing semiconducting photovoltaic systems, which potentially lead to substantially lower costs than silicon^24^. Many recent studies indicated that FeS_2_ has been successfully applied in the photo-electronic devices with a photoresponse from near infrared (NIR) range^27^,^28^,^29^. Previous reports have demonstrated the successful fabrications of pyrite NC-based polymer hybrid solar cell^30^ and photodiode devices^31^,^32^ with a spectral response extended to near infrared (NIR) wavelengths. Also, we found that the catalytic activity of FeS_2_ nanocrystals (NCs) in dye-sensitized solar cell as a counter electrode showed comparable catalytic efficiency with traditional precious Pt electrode^33^. However, the photovoltaic devices based on the FeS_2_ materials are still lacking of photovoltaic response due to the highly conductive surface-related defects in pyrite^34^,^35^. Although several recent reports indicated the FeS_2_ film was employed as a photoanode in PEC, the results showed the limited photoresponse in the visible light^28^,^36^. Therefore, it is still a great challenge to explore a new PEC photoanode using FeS_2_ materials with enhanced photocurrent response and extended light response to near infrared (NIR) range.

In this study, the photoanode consisting of earth abundant FeS_2_ formed on TiO_2_ thin film (FeS_2_/TiO_2_) for PEC applications was successfully prepared. The structure of FeS_2_/TiO_2_ photoanode was carefully characterized by high resolution scanning electron microscopy, transmission electron microscopy, powder X-ray diffraction and Raman microscopy. Also, the photocurrent response of the photoanode was measured under AM 1.5 illumination and NIR laser (808 nm) irradiation. We found that the photoresponse of the photoanode showed strong dependency on the sulfur deficiency and surface defect of FeS_2_. With proper sulfurization treatment, the surface defect of the FeS_2_/TiO_2_ photoanode was reduced, which optimized the photocurrent response of the photoanode.

## Experimental Section

### Fabrication of FeS_2_ NCs

In brief, FeCl_2_ (189 mg), 1,2-hexadecanediol (384 mg), octadecene (30 mL), and oleic acid (OA) (12 mL) were mixed and subsequently reacted under N_2_ gas at 100 °C for 1 h to form the Fe–olei cacid complex. Subsequently, oleylamine (OLA) (15 mL) solution of sulfur (576 mg) was quickly injected into the solution. The resulting solution was heated to 240 °C and maintained for 1 h. After the solution was cooled to room temperature, a large amount of methanol was added to precipitate as-grown FeS_2_ NCs, followed by centrifugation. To obtain the FeS_2_ NCs solution with high solubility for film depositions, the as-grown FeS_2_ NCs were further purified by washing with ethanol, ethanol/chloroform (10/1 vol.), and methanol/chloroform (10/1 vol.). Subsequently, the larger NCs and any residual side products from the NCs suspension were removed by addition of chloroform, followed by centrifugation at 3500 rpm for 10 min. The resulting FeS_2_ NCs solution with high solubility and purification can be obtained.

### Fabrications of FeS_2_/TiO_2_ photoanode

First, the fluorine doped tin oxide (FTO) glass was cleaned sequentially by a neutral cleaner, water, acetone, and IPA, as the initial step. A compact layer of TiO_2_ was coated on the FTO substrate using a solution, consisting of titanium (IV) isopropoxide (TTIP, + 98%, 0.5 g) in 2-methoxy ethanol (1.5 g), not only to obtain a good mechanical contact between the FTO and the TiO_2_ film but also to isolate the contact between the FTO and the electrolyte. Another TTIP solution was then hydrolyzed to acquire the TiO_2_ in a media, containing 0.1 M HNO_3_, by adopting a sol-gel method. The thus obtained TiO_2_ solution was autoclaved through a hydrothermal process at 240 ^o^C for 12 h. By concentrating the autoclaved solution to 13 wt%, a paste of nanocrystalline TiO_2_ was obtained. In order to prevent the paste from cracking and to control the pore size of TiO_2_, 15 wt% of PEG corresponding to the amount of TiO_2_ was added to the paste. The TiO_2_ layer as a photoanode on FTO for PEC was prepared through the following procedure. The TiO_2_ paste prepared above was coated on the FTO glass by using a doctor-blade method. The thus coated FTO glass was annealed at 450 ^o^C for 30 min. After repeating such a coating and sintering, another layer of TiO_2_ containing light scattering particles of 300 nm was coated on the FTO glass, and the sintering was performed in the same way. A TiO_2_ with active area of 1.0 cm^2^ was dipped overnight in a solution, containing 0.3 mM FeS_2_ solution in cholorform to from as-grown FeS_2_/TiO_2_ photoanode on FTO. Finally, The as-grown FeS_2_/TiO_2_ bilayer was further annealed by sulfur vapor at 450^o^ C for 3hr to form the FeS_2_/TiO_2_ photoanode.

### Electrochemical Measurement

Photoelectrochemical cell measurement was was carried out in a solution containing 0.35 M Na_2_SO_3_ and 0.24 M Na_2_S (pH = 13) with a standard three-electrodes system controlled by a Autolab electrochemistry workstation. The FeS_2_/TiO_2_ photoanode was used as working electrode, graphite rod as counter electrode and Ag/AgCl as reference electrode. The reference was calibrated against and converted to reversible hydrogen electrode (RHE). A AM 1.5 irradiation (100 mW/cm^2^, Newport Inc.) and a NIR continuous laser (808 nm) was used as the light source. Linear sweep voltammetry was carried out at 1 mV/s for the polarization curves.

### Characterizations

High-resolution Transmission Electron Microscopy (TEM) (HR-TEM) images were obtained using a Philips Technai G2 (FEI-TEM) microscopy operating at 200 kV. X-ray Diffraction (XRD) measurements were performed by Bruker D8 tools advance, operating with Cu Kα radiation (λ = 1.5406 Å) generated at 40 keV and 40 mA. Scans were done at 0.01 S^−1^ for 2θ value between 20° and 60°. UV-Vis-NIR absorption spectra were obtained using a Cary 500 UV-Vis-NIR spectrophotometer. The inductively coupled plasma atomic emission spectroscopy (ICP-AES) was used to measure the atomic ratio of FeS_2_ NCs. The external quantum efficiencies (EQEs) were measured by using a Xe lamp in combination with a monochromator (Oriel Inc.). A UV filter was also used to avoid the overtones of the monochromator’s grating from illuminating the specimen.

## Results and Discussion

**Figure 1:**
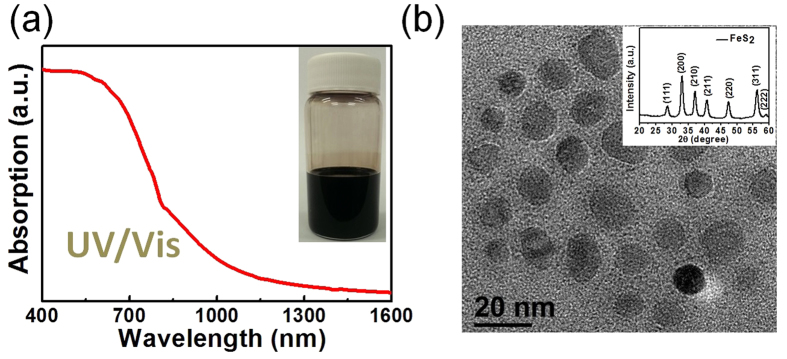
(**a**) UV-Vis-NIR absorption spectrum of FeS_2_ NCs. The inset showed the photograph image of the FeS_2_ NCs solution. (**b**) TEM image of FeS_2_ NCs. The average sizes of the NCs are calculated to be ∼15 nm. The inset showed the x-ray diffraction (XRD) pattern of the FeS_2_ NCs.

The FeS_2_NCs were prepared using wet solution-phase chemical syntheses with some modifications according to previous reports^31^,^37^. Figure 1 (a) demonstrated the UV–Vis–NIR absorption spectrum of FeS_2_ NC solution in chloroform. The absorption was extended to NIR wavelength ranging from 400 nm to 1300 nm. The inset of Fig. 1(a) showed the photograph image of the FeS_2_ NC solution, which was utilized to fabricate as grown FeS_2_/TiO_2_ photoanode by dip-coating process, as discussed in the experimental sections. Figure 1 (b) shows the high-resolution transmission electron microscopy (HR-TEM) image of FeS_2_ NCs with an average diameter of 15 nm. The inset of Fig. 1(b) showed the X-ray diffraction (XRD) pattern of the FeS_2_ NCs. The diffraction peaks were indexed to the (111), (200), (210), (211), (220), and (311) planes of pyrite cubic phase (JCPDS no. 42–1340). No other significant diffraction peak was observed in Fig. 1(b), indicating that the FeS_2_ materials on TiO_2_ film exhibited a single-phased pyrite structure.

For the fabrication of as grown FeS_2_/TiO_2_ photoanode, a paste of nanocrystalline TiO_2_ was first formed on the conductive FTO glass (TiO_2_/FTO) by the casting process. Then, FeS_2_ NC solution (80 mg/mL) was dip-coating onto TiO_2_/FTO substrates to form as-grown FeS_2_/TiO_2_ film on FTO substrate. Finally, to increase the crystallinity of FeS_2_ and reduce the interface connection, the as-grown FeS_2_/TiO_2_ film was sulfurized under sulfur vapor at a temperature of 450 ^o^C to form the resulting FeS_2_/TiO_2_ photoanode.

**Figure 2:**
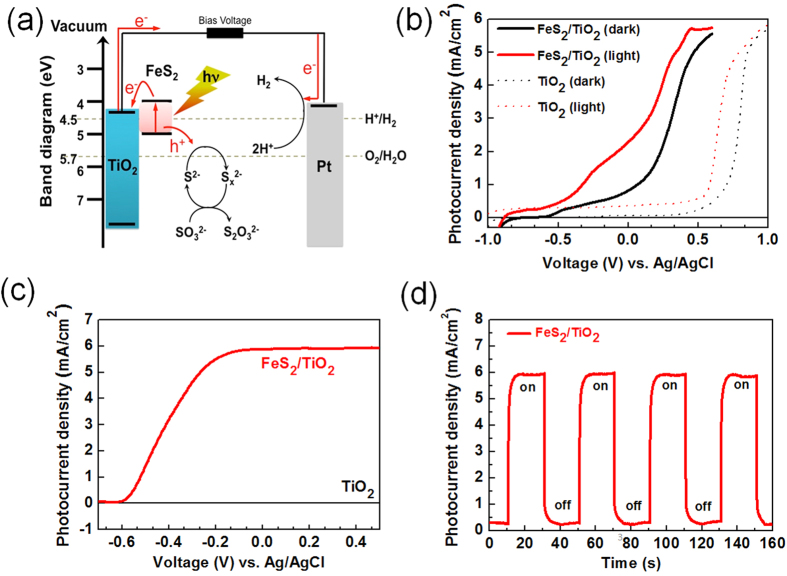
(**a**) Schematic illustration of the PEC device with a FeS_2_/TiO_2_ photoanode, and a passive Pt cathode, for light driven water splitting in aqueous solution. (**b**) The photocurrent–potential (I–V) responses of FeS_2_/TiO_2_ photoanode and pure TiO_2_ in the alkaline electrolyte (pH = 13.5) with SO_3_^2−^/S_2_O_3_^2−^ as sacrificial agent under simulated AM 1.5 illumination (100 mW/cm^2^). (**c**) The photocurrent–potential (I–V) responses of FeS_2_/TiO_2_, and TiO_2_ photoanodes in the alkaline electrolyte (pH = 13.5) with SO_3_^2−^/S_2_O_3_^2−^ as sacrificial agent under NIR laser (808 nm) illumination (300 mW/cm^2^). (**d**) Light chopping photocurrent measurements in a three electrode cell using FeS_2_/TiO_2_ photoanode as working electrode.

To test photoresponse behavior of the FeS_2_/TiO_2_ photoanode, PEC device (Fig. 2(a)) were carried out using the FeS_2_/TiO_2_ photoanode, a Pt wire cathode, and a Ag/AgCl reference electrode in the alkaline electrolyte (pH = 13.5) with SO_3_^2−^/S_2_O_3_^2−^ as sacrificial agent under simulated AM 1.5 illumination (100 mW/cm^2^) and NIR 808 nm laser (300 mW/cm^2^), respectively. The relevant energetics of each components obtained from related literature^15^,^31^. The band gap of FeS_2_ is located around 4.0 to 4.95 eV versus vacuum energy, which is similar to our previous report^31^. The formation of the FeS_2_/TiO_2_ photoanode with a satisfied energy-level alignment was expected to assist charge separation of photogenerated carriers. Briefly, when incoming light excites free electrons and holes near the surface of the FeS_2_ electrode, the electrons and holes were separated from TiO_2_ as an electron acceptor layer. The electrons flowed through the TiO_2_ layer to the cathode electrode at the other side (Pt electrode) of the cell, where generated the hydrogen gas during water reduction reaction. The holes react with the sacrificial agent (SO_3_^2−^/S_2_O_3_^2−^) in the electrolyte which can suppress photocorrosion of metal sulfide materials^38^.

Figure 2 (b) displays the current–voltage (*I–V*) curves of the FeS_2_/TiO_2_ photoanode and pure TiO_2_ photoanode under darkness and AM1.5 simulated sunlight, respectively. The current of FeS_2_/TiO_2_ photoanode could be determined at −0.6 V versus Ag/AgCl under darkness at first, then the current rises slowly to 1 mA/cm^2^ at 0.15 V versus Ag/AgCl. The current of FeS_2_/TiO_2_ photoanode found at −0.6 V represented that FeS_2_ exhibited a catalytic activity for the sacrificial agent. When the FeS_2_/TiO_2_ electrode was illuminated under AM1.5 illumination, the current was increased and reached 2-fold of the dark current at 0.15 V. This result indicated that the FeS_2_/TiO_2_ photoanode exhibited a photoresponse under AM-1.5. In order to distinguish the photoresponse contribution from FeS_2_ and/or TiO_2_, the PEC devices were illuminated under the NIR laser (808 nm) with 300 mW/cm^2^ for the comparison. Figure 2 (c) showed the *I*−*V* characteristics of the device. The results showed that the anodic photocurrents of FeS_2_/TiO_2_ photoanode increased as the potential was around −0.61 V, and reached saturation (5.8 mA/cm^2^) when the potential was higher than −0.2 V (vs Ag/AgCl). The photocurrent of the pure TiO_2_ photoanode is negligible under NIR illumination, indicating that FeS_2_ is a major contributor to the observed photocurrent under NIR illumination. Figure 2 (d) demonstrated the current-time (*i-t*) characteristics of the FeS_2_/TiO_2_ photoanode under the on/off cycles of the NIR illumination at a constant bias of 0.1 V. The results indicated that the photocurrent of the FeS_2_/TiO_2_ photoanode reached saturation very fast, representing the less surface traps in the FeS_2_ film during sulfurization treatment.

**Figure 3:**
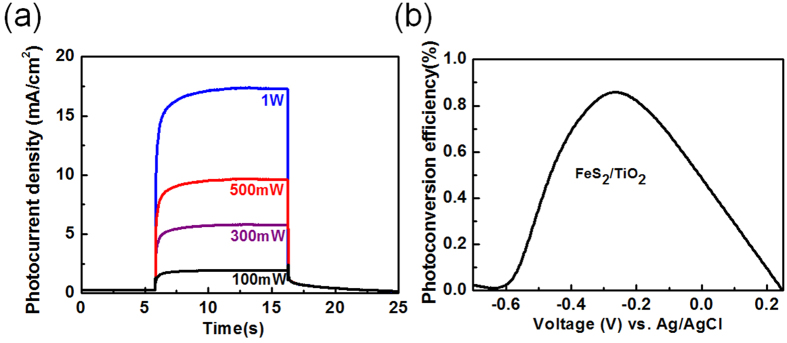
(**a**) The dependence of photocurrent of the FeS_2_/TiO_2_ photoanode operated at a bias of 0.1 V as a function of incident power under excitation with an 808 nm laser. (**b**) The photoconversion efficiency (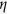) curves for the FeS_2_/TiO_2_ photoanode.

Figure 3 (a) represented the dependence of photocurrent of the FeS_2_/TiO_2_ photoanode operated at a bias of 0.1 V as a function of incident power under excitation with an 808 nm laser. The photocurrent of the photoanode exhibited a linear increase with incident power, which may be attributed to efficient carrier transport and collection of the FeS_2_ thin film between the heterjunction of FeS_2_ and TiO_2_ layers. The photoconversion efficiency (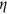) curves for the FeS_2_/TiO_2_ photoanodes are presented in Fig. 3(b). The photoconversion efficiency are calculated using the following equation,


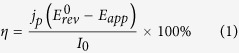


where 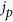 is photocurrent density, 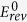 is standard state-reversible potential (i.e. 1.23 V vs. RHE), 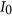 is the intensity of the incident light, and 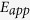 is the applied potential vs. RHE. At a bias of 0.7 V, the efficiency of FeS_2_/TiO_2_ photoanode reached~ 0.84% at NIR irradiation which was the highest efficiency in PEC measured to date for FeS_2_ materials^28^,^36^.

**Figure 4:**
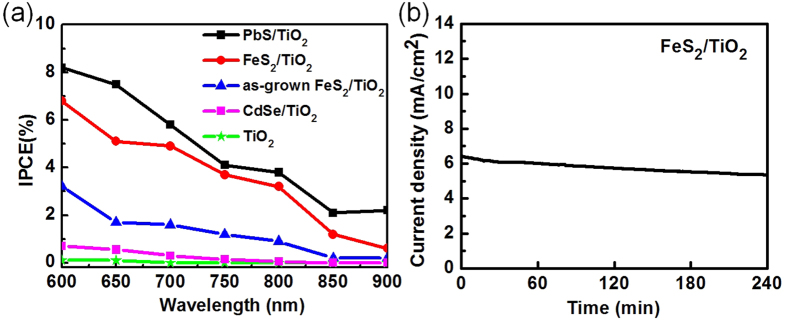
(**a**) Incident photon to current conversion efficiency of TiO_2_, CdSe/TiO_2_, as-grown FeS_2_/TiO_2_, FeS_2_/TiO_2_ and PbS/TiO_2_ photoanodes. (**b**) Stability test of the FeS_2_/TiO_2_ photoanode in the alkaline electrolyte (pH = 13.5) with SO_3_^2−^/S_2_O_3_^2−^ as sacrificial agent under NIR laser (808 nm) illumination (300 mW/cm^2^).

To further quantify the PEC performance, incident photon to current conversion efficiency (IPCE) measurements (Fig. 4(a)) have been made to study the photoresponse of the FeS_2_/TiO_2_ photoanode from visible light to NIR. For the comparison, CdSe/TiO_2_ and PbS/TiO_2_ photoanodes were both fabricated in this work. Their detailed synthesis, characterization and device fabrications were described in the supporting information. We found that the FeS_2_/TiO_2_ photoanode and PbS/TiO_2_ photoanode showed higher photoresponse in the NIR region than that of as grown FeS_2_/TiO_2_ photoanode and CdSe/TiO_2_ photoanode. No photoresponse of the pure TiO_2_ photoanode was found from wavelength of 600 nm to 900 nm due to its large band gap of 3.7 eV. Moreover, the IPCE value of FeS_2_/TiO_2_ photoanode at illumination light from 600 nm to 900 nm is improved~ 2-fold in comparison with as-grown FeS_2_/TiO_2_. The stability of the FeS_2_/TiO_2_ photoanode was studied by a chronoamperometric (*i*–*t*) measurement (Fig. 4(b)). Under NIR light irradiation, the clearing of bubbles found at cathode electrode in our PEC device. The results of *i*–*t* measurement showed that the photocurrent of the FeS_2_/TiO_2_ photoanode remained stable over continuous operation for 4 hours under alkaline conditions at a bias of 0.1 V versus Ag/AgCl reference. The retention of both photoanodes exceeded 80%. In comparison with other metal sulfide case, our PbS/TiO_2_ photoanode was not stable and their retention is only 50% under operation of 4hr which is similar to other report^39^.

**Figure 5:**
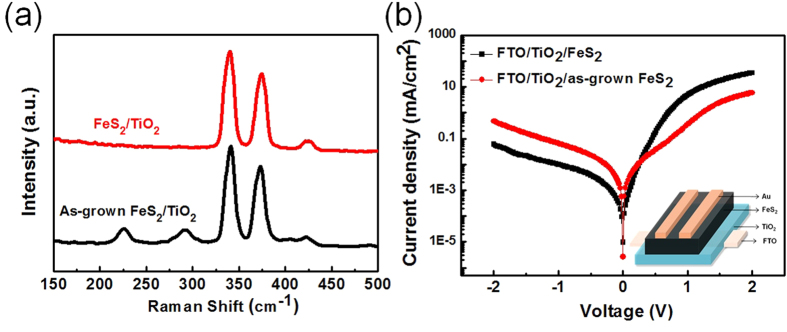
(**a**) Raman spectra of as-grown FeS_2_/TiO_2_ photoanode and FeS_2_/TiO_2_ photoanode. (**b**) The dark current of TiO_2_/ as-grown FeS_2_ and TiO_2_/FeS_2_ devices.

To eliminate the surface defects of as-grown FeS_2_/TiO_2_ film, the sulfurization process was carried out to reduce the sulfur deficiency in the FeS_2_ film. The change of sulfur deficiency of FeS_2_ NCs before and after annealing was analyzed by inductively coupled plasma atomic emission spectroscopy (ICPAES). The ratio of Fe to S in the as-grown FeS_2_ thin film is 1:1.92 and the sulfur deficiency is approximately 4.5% higher than ~ 1.94–1.98 for the FeS_2_ film after sulfurization based on ICPAES measurement. Furthermore, in Raman spectra (Fig. 5(a)), we found that there are only three peaks found at 343, 379, and 430 cm^−1^ in the FeS_2_/TiO_2_ photoanode with sulfurization treatment, which are the characteristic active modes for pure pyrite corresponding to the S_2_ libration (Eg), S–S in-phase stretch (Ag), and coupled libration and stretch (Tg) modes, respectively. However, there is a few FeS phase (presence of Raman peaks at 210 and 280 cm^−1^) observed in the as-grown FeS_2_/TiO_2_ film. FeS phases could be caused by the sulfur deficiency on the surface of as-grown FeS_2_. Several previous reports indicated that there is less possibility to make the iron pyrite thin film as photoactive in photovoltaic device by using solution process because of lots of surface states and the sulfur vacancies^40^. The large short-circuit photocurrent densities have been only found from pyrite single crystals, which suffered from a low open-circuit voltage and low efficiency^31^,^40^. In our work, the FeS_2_ sensitized on TiO_2_ photoanode for the PEC application was successfully fabricated by a simple solution process. Besides, TiO_2_ played an important role in enhancing charge transfer between the interface of FeS_2_ and TiO_2_ to improve the photo conversion efficiency of the FeS_2_/TiO_2_ photoanode. Figure 5(b) showed the dark current of TiO_2_/ as-grown FeS_2_ and TiO_2_/FeS_2_ devices. We found that TiO_2_/FeS_2_ device exhibited a better rectification ratio than that of TiO_2_/as-grown FeS_2_ device. Forward bias current was enhanced and reverse bias current was also reduced by an order, indicating that TiO_2_/FeS_2_ device with reducing the sulfur vacancies substantially improved pn junction behavior with a clearly rectifying current-voltage characteristic in comparison with TiO_2_/ as-grown FeS_2_ device. Therefore, overall results indicated that our FeS_2_/TiO_2_ photoanode have achieved a high photocurrent response extended from visible light to NIR range (900 nm) in PEC, leading to H_2_ generation successfully in the cathode electrode.

## Conclusions

This study demonstrated that the FeS_2_/TiO_2_ photoanode composed of all earth-abundant elements exhibited high photo response from visible to NIR range for PEC hydrogen generation. The surface defect of FeS_2_ was found to be a critical factor to affect the photo response of FeS_2_/TiO_2_ photoanode in PEC application. The proper sulfurization was utilized to eliminate surface defect of FeS_2_ and to enhance the interface charge transfer between FeS_2_ and TiO_2_. We believed that this work demonstrated not only a breakthrough of using FeS_2_ as photoanode materials to generate hydrogen from the input of visible to NIR radiation but also a new approach for the design of sulfur-based binary compounds for photoelectrochemical applications.

## Additional Information

**How to cite this article**: Wang, D.-Y. *et al.* Iron Pyrite/Titanium Dioxide Photoanode for Extended Near Infrared Light Harvesting in a Photoelectrochemical Cell. *Sci. Rep.* **6**, 20397; doi: 10.1038/srep20397 (2016).

## Supplementary Material

Supplementary Information
