# Supplementary material for: Iron Pyrite/Titanium Dioxide Photoanode for Extended Near Infrared Light Harvesting in a Photoelectrochemical Cell
Source: Sci Rep. 2016 Feb 8;6:20397. doi: 10.1038/srep20397 (PMC4745049; doi:10.1038/srep20397)
Supplement: Supplementary Information [file srep20397-s1.pdf]

Supporting Information

Iron Pyrite/Titanium Dioxide Photoanode for Extended Near Infrared Light Harvesting in a Photoelectrochemical Cell

Di-Yan Wang,[1*] Cheng-Hung Li,[1] Shao-Sian Li,[2] Tsung-Rong Kuo,[1] Chin-Ming Tsai,[1] Tin-Reui Chen,[1] Ying-Chiao Wang,[2] Chun-Wei Chen,[2*] Chia-Chun Chen,[1,3*]

a. Synthesis of PbS nanocrystals.

The PbS nanocrystals (QDs) were prepared by the wet solution phase chemical synthesis with some modifications. In brief, 223 mg of PbO and 10 ml of oleic acid were mixed and then were allowed to react under N_2 gas at 150 ^oC for 60 min to form the Pb–oleic acid complex. Afterwards, 2.5 ml 1-octadecene solution of bis(trimethylsilylmethyl) sulfide (96 mg) was quickly injected into the solution. The resulting solution was heated to 220 ^oC and kept for 60 min. Two washing steps are included to remove un-reacted materials and solvents. After the solution was cooled to room temperature, a large amount of methanol was added to precipitate PbS QDs followed by centrifugation. The solid product was dispersed well in chlorobenzene.

b. Synthesis of CdSe nanocrystals.

0.012 g of CdO (0.1 mmol), and 0.114 g of stearic acid (0.4 mmol) were loaded into a 25 mL three-neck flask and heated to 150 ^oC under N_2 flow until CdO was completely dissolved. The color of solution changed from brown to colorless. The mixture was allowed to cool to room temperature. TOPO and hexadecylamine (HDA), 1.94 g of each, were added to the flask, and the mixture was heated to 250 ^oC under

N_2 flow to form an optically clear solution. When the temperature was stable, the Se solution containing 0.079 g (1 mmol) of Se dissolved in 2 mL of TBP was swiftly injected into the reaction flask. After the injection, the temperature was set at 180 ^{o}C for growth of the nanocrystals and kept about 1 h. Then, the reaction solution was cooled to room temperature. The reaction solution was mixed with chloroform then centrifuged to remove insoluble solid. The CdSe nanocrystals were precipitated by adding methanol into the chloroform solution and isolated by centrifugation. The resulting precipitate was stored in N_2 for future use.

c. Preparation of PbS/TiO_2 photoanode and CdSe/TiO_2 photoanode.

A TiO_2 with active area of 1.0 cm^2 was dipped overnight in a solution, containing 0.3 mM PbS and CdSe solution in cholorform to from PbS_2/TiO_2 photoanode and CdSe/TiO_2 photoanode on FTO, respectively.

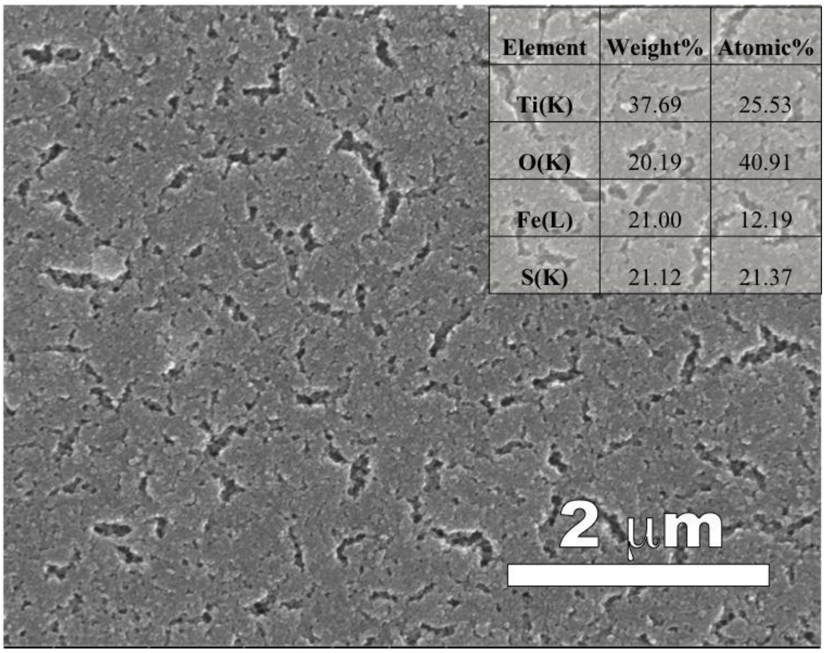

Figure S1. Scanning electron microscopy image of FeS_2/TiO_2 photoanode with their atomic composition.

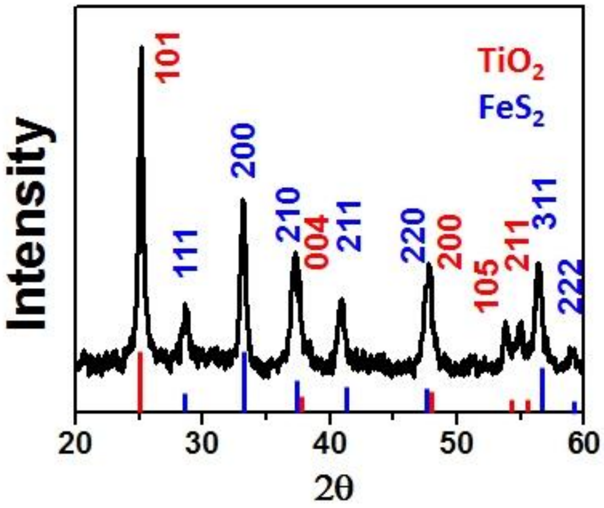

Figure S2. XRD spectrum of FeS_2-TiO_2 photoanode, including cubic iron pyrite phase (blue mark, JCPDS File No. 42-1340) and anatase TiO_2 phase (red mark, JCPDS File No. 21-1272).

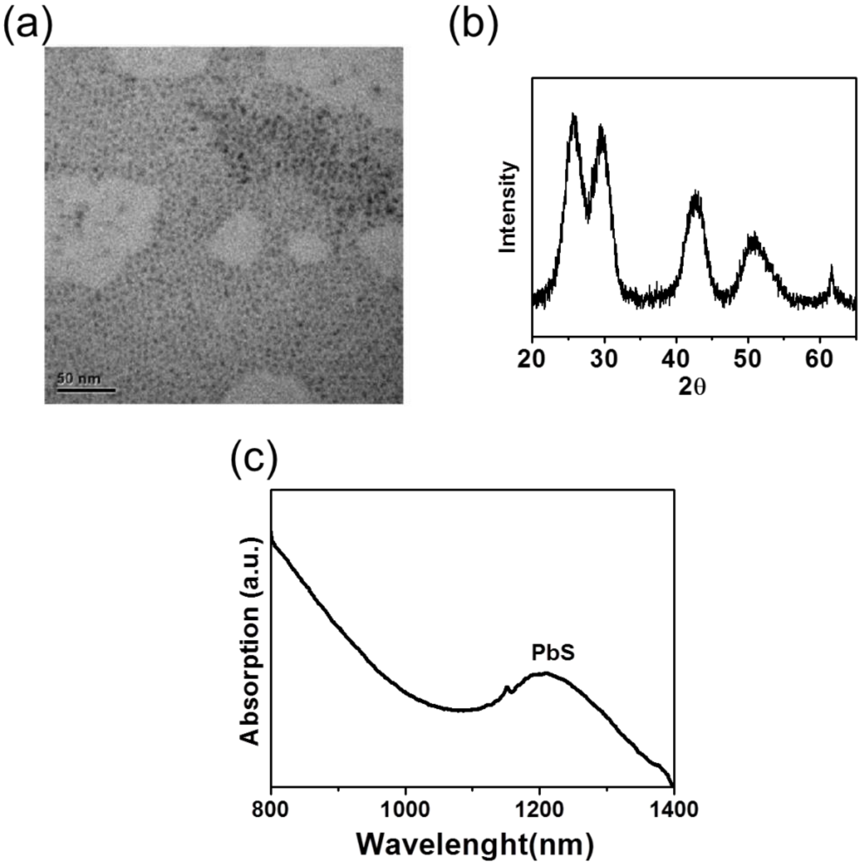

Figure S3. (a) TEM image, (b) XRD spectra and (c) UV/Vis spectra of PbS nanoparticles.

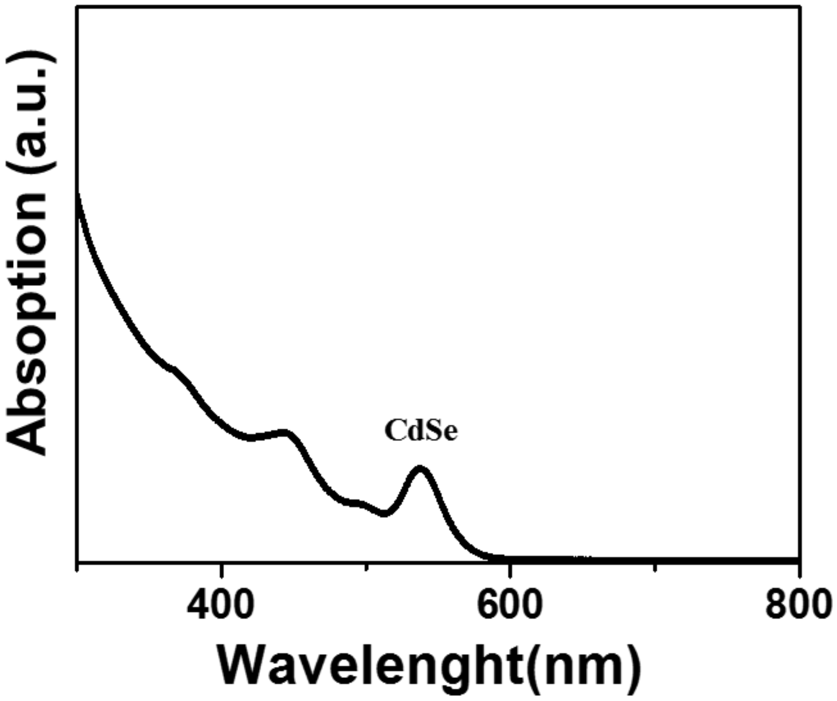

Figure S4. UV/Vis spectrum of CdSe nanoparticles.
